# Genetic diversity and genotyping of *Echinococcus multilocularis*: a minireview

**DOI:** 10.3389/fpara.2025.1721690

**Published:** 2025-12-05

**Authors:** Franziska Rachel, Franz Josef Conraths, Pavlo Maksimov

**Affiliations:** 1 Friedrich-Loeffler-Institut – Federal Research Institute for Animal Health (FLI), Institute of Epidemiology, National Reference Laboratory for Echinococcosis, Greifswald, Germany; 2 Department of Biology, Faculty of Mathematics and Natural Sciences, University of Greifswald, Greifswald, Germany; 3 Surveillance Authority for Public Law Duties of the Medical Service East, Bundeswehr, Potsdam, Germany

**Keywords:** *Echinococcus multilocularis*, genotyping, genetic diversity, methods, minireview

## Abstract

The genome of *Echinococcus multilocularis*, one of the most dangerous endoparasites for humans in the northern hemisphere, has been studied for decades, but its global genetic diversity has not yet been fully deciphered. Yet, our understanding of the diversity of this parasite has recently improved significantly due to the development of new genotyping methods. However, the use of different methods and markers has made it difficult—and in some cases impossible—to compare existing studies directly. As a result, accurate information on the global genetic diversity of *E. multilocularis* remains unavailable, although such knowledge is essential from both clinical and epidemiological perspectives. Here we provide an overview of the state of knowledge on the genetic diversity of *E. multilocularis*, and the methods used for genotyping this parasite and provide an outlook on needed future research to understand the diversity of this fascinating parasite.

## Introduction

*Echinococcus* (*E*.) *multilocularis* (Leuckart, 1863; Vogel, 1957; Vuitton et al., 2020) is a dangerous and zoonotic endoparasite of the northern hemisphere (Rausch, 1967; Conraths et al., 2017; Deplazes et al., 2017; Thompson, 2017; Vuitton et al., 2020) causing alveolar echinococcosis (AE), which can be fatal if left untreated (Vuitton et al., 2015). Even with adequate treatment it is a chronic and serious disease that can cause long-term physical and psychosocial stress for affected people (Vuitton et al., 2015, Vuitton et al., 2020; Autier et al., 2022; Nikendei et al., 2023; Antolová et al., 2024).

Genetic studies have significantly changed our understanding of the diversity of this parasite in recent years. At the end of the 20th and beginning of the 21st century, it was still assumed that the genetic diversity of *E. multilocularis* was rather low or that the existing genetic markers were insufficient to study genetic diversity in sufficient depth (Bowles et al., 1992; Bart et al., 2003; Nakao et al., 2009). In recent years, a significantly higher variability in the genome of *E. multilocularis* could be detected with the arrival of new genotyping methods (Nakao et al., 2009; Šnábel et al., 2020; Bohard et al., 2023; Lallemand et al., 2024; Santoro et al., 2024; Rachel et al., 2025).

Investigating the diversity of *E. multilocularis* is important, because knowledge on different genotypes can contribute to a better understanding of the spread, genetic adaptation, and virulence of the parasite in different hosts. Moreover, detailed knowledge on the genetic diversity may help to establish more targeted diagnostics, to improve the tracing back of the infection source, and to develop better therapeutic strategies (Wen et al., 2019; Casulli and Tamarozzi, 2021; Romig and Wassermann, 2024; Simoncini and Massolo, 2024). All this can also help to improve our understanding of the epidemiology of this parasite. The aim of this minireview is therefore to present a concise overview and a summary of the current state of knowledge regarding the genetic diversity and genotyping of *E. multilocularis*, with the goal of identifying promising methods and future opportunities in this field.

## Taxonomy, life cycle, and distribution

**Figure 1:**
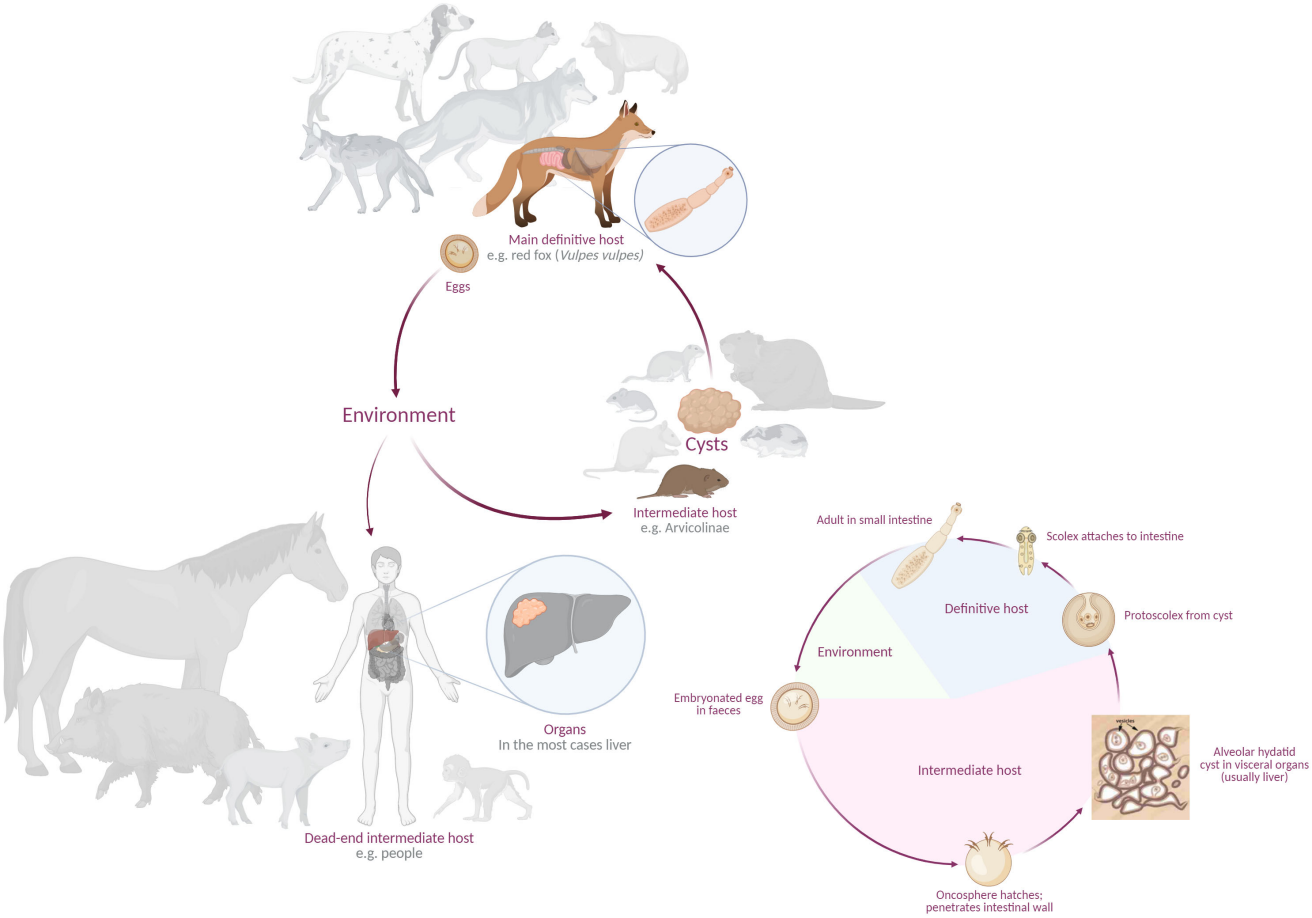
Life cycle of *Echinococcus multilocularis* with a selection of definitive, intermediate and dead-end intermediate hosts, as well as all stages of development of the parasite (created in BioRender. Rachel, F. (2025)https://BioRender.com/q81lfds and modified after Thompson (2017) and Centers for Disease Control and Prevention (2019)).

*E. multilocularis* (Leuckart, 1863) is a species of the genus *Echinococcus* that occurs in the northern hemisphere (Oksanen et al., 2016; Simoncini and Massolo, 2024). Various rodents and lagomorphs are involved in the life cycle as intermediate hosts, depending on the spatial distribution of the respective species in the geographic area where the parasite is endemic. Humans are dead-end intermediate hosts and various canids serve *E. multilocularis* as definitive hosts (**Figure 1**) (Woolsey and Miller, 2021; Romig and Wassermann, 2024; Lundström-Stadelmann et al., 2025). In Europe, the sylvatic life cycle of *E. multilocularis* dominates, but there is also a ‘domestic life cycle’, in which dogs play a central role. Both cycles can lead to an accidental oral infections of humans. Infections with cats as definitive hosts have been reported, but the prevalence is usually low and their role in the life cycle has been controversially discussed due to the usually low intensity of infection (Petavy et al., 2000; Dyachenko et al., 2008; Umhang et al., 2015; Knapp et al., 2016; Karamon et al., 2019; Umhang et al., 2022) and the limited number of excreted eggs (Thompson et al., 2006; Dyachenko et al., 2008; Umhang et al., 2015, Umhang et al., 2022). One study showed that eggs shed by cats led to a few infections in mice (Kapel et al., 2006). Therefore, the risk of humans infections through eggs shed by cats seems rather low (Thompson et al., 2006; Hegglin and Deplazes, 2013; Umhang et al., 2015; Oksanen et al., 2016), indicating that cats play a minor role in the epidemiology of the parasite (Thompson et al., 2006; Hegglin and Deplazes, 2013; Conraths and Deplazes, 2015; Umhang et al., 2015; Oksanen et al., 2016). In conclusion, cats seem to play a negligible role in transmission (Kamiya et al., 1985; Thompson et al., 2003; Umhang et al., 2015; Knapp et al., 2016; Furtado Jost et al., 2023). Studies on the spread of *E. multilocularis* show that, in addition to the presence of specific hosts, the prevalence is highly likely to be influenced by environmental factors such as temperature and humidity, as well as by the behaviour of hosts (Schneider et al., 2023) and land-use (Staubach et al., 2001).

The taxonomy of the genus *Echinococcus* has undergone a transformation since the diseases caused by different species of this genus were first described. While Virchow (1855) only distinguished the two species *E. multilocularis* and *E. granulosus* on the basis of the clinical presentation of the respective diseases these parasites caused, Leuckart (1863) described *E. multilocularis* as a separate species. The years that followed until the late 20th century were marked by ambiguous classifications and taxonomic confusion, which led, for example, to postulating subspecies of *E. multilocularis*, which are no longer recognised (Romig et al., 2017; Thompson, 2020; Vuitton et al., 2020). Genetic classification of the members of the genus *Echinococcus* into four distinct species only became possible in the early 1990s (Bowles et al., 1992). Nevertheless, doubts regarding the validity of some species arose over time due to results of the first genetic studies, as the phenotype of the adult parasite in different hosts did not always reflect the found genotype (Bowles and McManus, 1993a). In the following years, the examination of isolates from different continents (North America, Europe, Asia) revealed a clear genetic differentiation of populations of *E. multilocularis* (Šnábel et al., 2020). The species *E. multilocularis* could thus be clearly separated from the *E. granulosus sensu lato* (*s.l.*) complex, but despite substantial progress, it was still difficult to classify the genus *Echinococcus* taxonomically and phylogenetically in a correct fashion (Knapp et al., 2015). Molecular biology proved invaluable in resolving this issue, as it not only reinstated the original taxonomic hypotheses, but also confirmed the reliability of distinct morphological characteristics by incorporating sequence data (Thompson, 2020). In addition to *E. multilocularis*, nine other species in the genus *Echinococcus* could be genetically confirmed or identified (*E. granulosus*, *E. equinus*, *E. ortleppi*, *E. canadensis*, *E. intermedius*, *E. felidis*, *E. shiquicus*, *E. vogeli*, *E. oligarthra*) (Thompson, 2020; Vuitton et al., 2020).

Today *E. multilocularis*, measuring only 2 to 4 mm in size, with four suction cups on its head, a double hook crown, and usually five proglottids, is classified in the phylum Platyhelminthes, class Cestoda, subclass Eucestoda, family Taeniidae, and genus *Echinococcus*, along with nine other species (Nakao et al., 2010b; Thompson, 2017; Conraths and Maksimov, 2020; Vuitton et al., 2020).

## Genetic diversity of *Echinococcus multilocularis* – state of research

**Figure 2:**
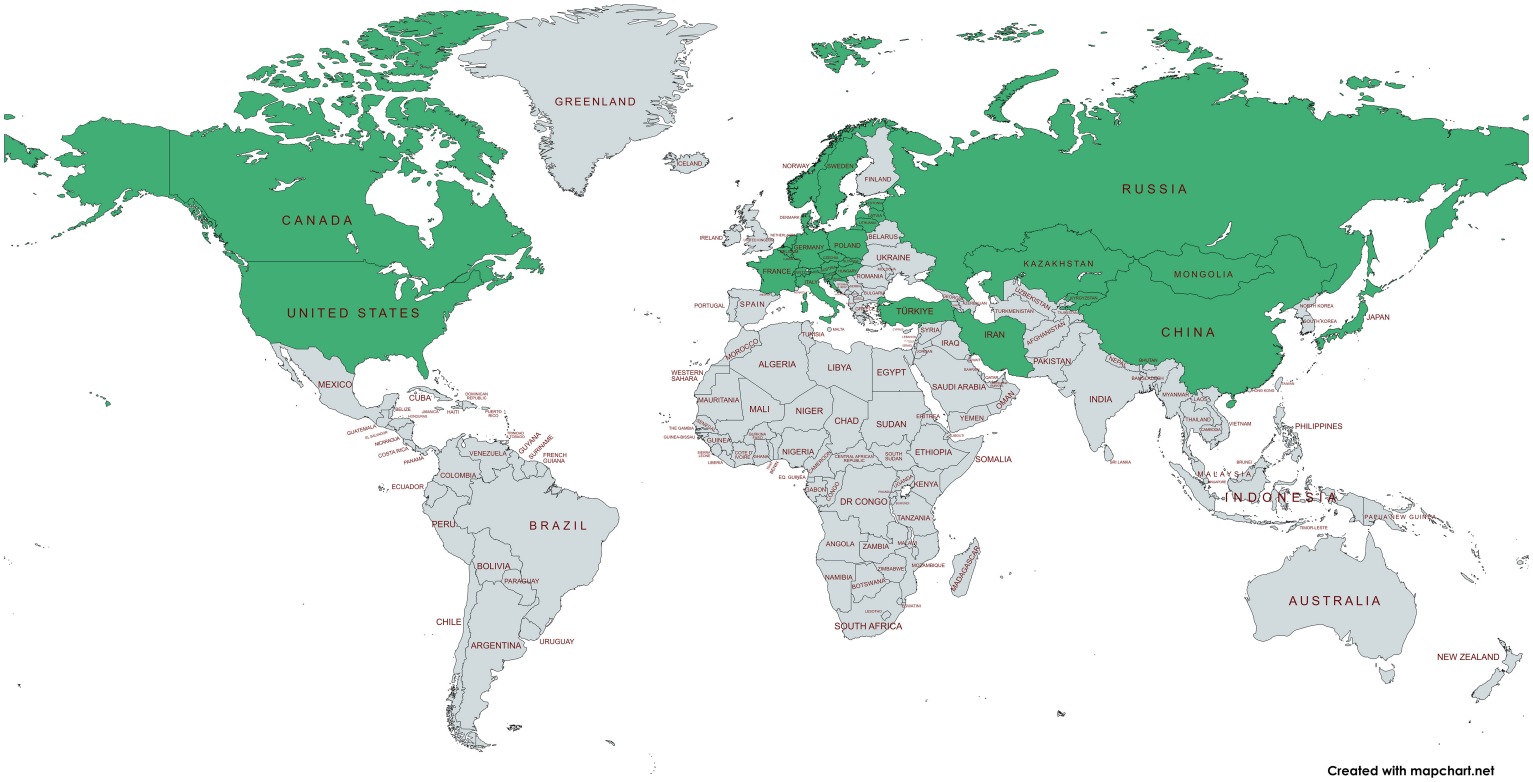
World map showing the countries (in green) in the northern hemisphere that were examined in the papers included in this minireview (created with mapchart.net).

The genetic diversity of *E. multilocularis* is the focus of scientific interest as the infection is a typical example for the One Health approach, as animals and humans can be infected, environmental factors are important for transmission, and it is suspected that different genotypes may have different virulence (**Table 1** and **Figure 2**) (Vogel et al., 1990; Bowles et al., 1992; Nakao et al., 2009; Spotin et al., 2018; Herzig, 2019; Šnábel et al., 2020; Bohard et al., 2023; Conlon et al., 2024; Cafiero et al., 2025; Guo et al., 2025; Rachel et al., 2025). The researchers’ questions focus on the following areas: evolutionary development, taxonomy, geographic spread across the Northern Hemisphere over time, virulence in various hosts with a particular focus on humans due to severe alveolar echinococcosis, and molecular epidemiology (**Table 1**).

**Table 1:** Overview of scientific papers published since 1990 (up to January 2025) on the topic of genetic diversity in *Echinococcus multilocularis*.

Paper	Geographic origin of samples	Animal	Analysis method(s)	Genotype/Haplotype/Profile	Gene/Marker	Fragment length of the gene [bp]	Accession no.
Vogel et al. (1990)	Switzerland, France, Canada, United States Germany	Human (*Homo sapiens*), common vole (*Microtus arvalis*)	Cloning via plasmid	–	pAL 1	600	–
Bowles et al. (1992)	China, North America, Europe (Germany)	Human (*Homo sapiens*), naturally infected rodent	Sequencing	M1, M2	*cox1*	366 of 1,608	–
Bowles and McManus (1993a)	China, North America, Europe (Germany)	Human (*Homo sapiens*), naturally infected rodent	Sequencing	M1, M2	*nad1*	471 of 894	–
Okamoto et al. (1995)	Japan, United States	Gray red-backed vole (*Clethrionomys rufocanus bedfordiae*), pig (*Sus domesticus*), Norway rat (*Rattus norvegicus*), tundra vole (*Microtus oeconomus*)	Sequencing	–	*cox1*	391 of 1,608	–
Bretagne et al. (1996)	France, United States, Germany, Switzerland, Japan	Common vole (*Microtus arvalis*), muskrat (*Ondatra zibethicus*), human (*Homo sapiens*), water vole (*Arvicola terrestris*), hispid cotton rat (*Sigmodon hispidus*), grey red-backed vole (*Craseomys rufocanus*), tundra vole (*Microtus oeconomus*), red fox (*Vulpes vulpes*)	Microsatellite	A profile, B profile, C profile	U1 snRNA genes	1,300 of 1,300	–
Haag et al. (1997)	Switzerland, Canada, United States, Japan, France, Germany	Human *(Homo sapiens*), monkey, rodent, red fox (*Vulpes vulpes*)	Sequencing	Genotype A & Genotype B	*nad1*, AgB/1, ActII, Hbx2	*nad1* (141 of 894), AgB/1 (102), ActII (268), Hbx2 (331)	L07774, AF003748, AF003749, AF003750, X66818, AF003976, AF003977, AF003978, Z26481, Z26482, Z26483, U65748
Rinder et al. (1997)	Germany, Japan, United States	Red fox (*Vulpes vulpes*), vole (*Clethrionomys rufocanus bedfordiae*), human (*Homo sapiens*), vole (*Microtus oeconomus*), vole (*Clethrionomys rutilus*)	Cloning and sequencing	Genotype 1 & Genotype 2	ITS1, ITS2, and adjoining rRNA coding regions	1300	–
von Nickisch-Rosenegk et al. (1999)	Austria, France, Germany, USA	*Microtus arvalis*, *Meriones unguiculatus*	Sequencing	–	*rrnS*	304	–
van Herwerden et al. (2000)	United States, Eurasia	Human (*Homo sapiens*), red fox (*Vulpes vulpes*)	Cloning and sequencing	M1	ITS1	934, 619	–
Nakao et al. (2003)	Japan	Red fox (*Vulpes vulpes*)	Microsatellite	Different alleles of EMms1, Emms2	EMms1, Emms2	864 (EMms1), 894 (EMms2)	AB100031.1, AB100032.1
Bart et al. (2006)	United States, Canada, Switzerland, France, Czech Republic, Austria	Vole (*Microtus oeconomus*), human (*Homo sapiens*), water vole (*Arvicola terrestris*), Ouistiti (Callitrichide´s), Cercopitheque, red fox (*Vulpes vulpes*)	Cloning and microsatellite	–	EmsB	200	–
Okamoto et al. (2007)	Japan, United States	Gray red-backed vole (*Clethrionomys rufocanus bedfordiae*), pig (*Sus domesticus*), Norway rat (*Rattus norvegicus*), tundra vole (*Microtus oeconomus*)	Sequencing and DNA fingerprinting	–	*cox1*, (CAC)_5_ probe	391 of 1,608 (*cox1*)	–
Knapp et al. (2007)	United States, Canada, China, Japan, France, Switzerland, Germany, Austria, Poland, Czech Republic, Slovakia, Netherlands	Tundra vole (*Microtus oeconomus*), human (*Homo sapiens*), Rodent, lacustrine vole (*Microtus limnophilus*), Kam Dwarf hamster (*Cricetulus kamensis*), red fox (*Vulpes vulpes*), water vole (*Arvicola terrestris*), macaque monkey, vervet monkey, marmoset monkey	Microsatellite	European (D, E, F, G, H)	EmsB, EmsJ, EmsK, NAK1	EmsB (209–241), EmsJ (152–155), EmsK (248–250), NAK1 (189–201)	AY680845, AY680857, AY680860, AB100031
Knapp et al. (2008)	France	Red fox (*Vulpes vulpes*)	Sequencing and microsatellite	different genotypes	EmsB, NAK1, *atp6*	EmsB (209–241), NAK1 (189–201), *atp6* (512 of 516)	EU044715, EU044717, EU044716
Bagrade et al. (2008)	Latvia	Red fox (*Vulpes vulpes*)	Sequencing	Europe	*cox1*, *nad1*, *rrnS*, *atp6*, *actII*	*cox1* (789 of 1,608), *nad1* (589 of 894), *atp6* (516 of 516), *rrnS* (362), *actII* (459)	–
Casulli et al. (2009)	Italy	Red fox (*Vulpes vulpes*)	Microsatellite	Four main genotypes	EmsB	EmsB (209–241)	–
Knapp et al. (2009)	Poland, France, Czech Republic, Switzerland, Germany, Austria, Slovakia	Red fox (*Vulpes vulpes*)	Microsatellite	G1-G32	EmsB	EmsB (209–241)	–
Nakao et al. (2009)	Austria, France, Germany, Belgium, Slovakia, Kazakhstan, United States, Japan, China	Laboratory strain, red fox (*Vulpes vulpes*), gerbils (*Meriones unguiculatus*), vole, human (*Homo sapiens*), domestic dog (*Canis lupus familiaris*)	Sequencing	Europe (E1-E5), Asia (A1-A10), North America (N1-N3), O1	*cob*, *cox1*, *nad2*	*cob* (1,068 of 1,068), *cox1* (1,608 of 1,608), *nad2* (882 of 882)	AB461395–AB461420 and AB477009– AB477012
Nakao et al. (2010a)	China	Human (*Homo sapiens*), rodent (*Microtus fuscus*, *Microtus limnophilus* and *Cricetulus kamensis*), hare (*Lepus oiostolus*), pika (*Ochotona curzoniae*)	Sequencing	M01 to M05 (for *cox1*)	*cox1* (mtDNA), *ef1a* (gDNA)	*cox1* (789 of 1,608), *ef1a* (656)	AB491414-AB491471
Casulli et al. (2010)	Hungary	Red fox (*Vulpes vulpes*)	Microsatellite	European (H, G, E, D)	EmsB	EmsB (209–241)	–
Knapp et al. (2012)	Norway	Sibling vole (*Microtus levis*)	Sequencing and microsatellite	EmsB (Svalbard)	EmsB, *elp*, *cal*, *th*	EmsB (209–241), *elp* (1023), *cal* (1368), *th* (583)	FR820773 - FR820783, FR820594 - FR820596
Ma et al. (2012)	China	Qinghai vole (*Microtus fuscus*), domestic dog (*Canis lupus familiaris*), human (*Homo sapiens*)	Sequencing	Haplotypes H16 and H17	*cox1*	*cox1* (792 of 1,608)	–
Konyaev et al. (2013)	Russian Federation	Human (*Homo sapiens*), narrow-headed vole (*Microtus gregalis*), tundra vole (*Microtus oeconomus*), gray red-backed vole (*Myodes rufocanus*), red fox (*Vulpes vulpes*), wolf (*Canis lupus*), flat-headed vole (*Alticola strelzowi*), lake Baikal mountain vole (*Alticola olchonensis*), arctic fox (*Vulpes lagopus*), Senegal bushbaby (*Galago senegalensis*)	Sequencing	Asia (EmRUS1-EmRUS12), Mongolia (EmRUS17, EmRUS18), North America (EmRUS13-EmRUS15), Europe (EmRUS16)	*cox1*	*cox1* (1,608 of 1,608)	AB688125, AB688134, AB777914, AB777915, AB777920, AB777921, AB777916, AB777917, AB777918, AB777919
Schurer et al. (2014)	Canada	Wolf (*Canis lupus*)	Sequencing	Europe (M2), european haplotype	*nad1*	*nad1* (395 of 894)	JX266826.1, AJ237640.1, JF751034.1
Umhang et al. (2014)	France	Red fox (*Vulpes vulpes*)	Microsatellite	P01-P22	EmsB	EmsB (209–241)	–
Gesy et al. (2014)	Canada	Coyote (*Canis latrans*), wolf (*Canis lupus*), arctic fox (*Vulpes lagopus*), deer mice (*Peromyscus maniculatus*)	Sequencing	Haplotypes A - Q	*nad1*	*nad1* (894 of 894)	KF962555 - KF962571
Gesy and Jenkins (2015)	Canada	Coyote (*Canis latrans*), deer mice (*Peromyscus maniculatus*)	Sequencing	SK1 - SK8	*cob*, *cox1*, *nad2*	*cob* (693 of 1,068), *cox1* (899 of 1,608), *nad2* (623 of 882)	KC549993, KC550008, KC582628-33, 550007, KC582621-26, KC549999, KC550006, KC582614-19
Karamon et al. (2017)	Poland	Red fox (*Vulpes vulpes*)	Sequencing	Asia (Haplotype EmPL9) & Europe (Haplotypes EmPL1-8 & EmPL10-15)	*cob*, *cox1*, *nad2*	*cob* (1,068 of 1,068), *cox1* (1,608 of 1,608), *nad2* (882 of 882)	KY205662–KY205706
Umhang et al. (2017)	Poland	Red fox (*Vulpes vulpes*)	Microsatellite	Pol01 - Pol29	EmsB	EmsB (209–241)	–
Abulizi et al. (2018)	China	Human (*Homo sapiens*)	Sequencing	H1 - H5 (*cox1*) and H1 - H5 (*cob*)	*cob*, *cox1*	*cob* (547 of 1,068), *cox1* (513 of 1,608)	new *cox1* no.: H4 (MF-370866) and H5 (MF370867); new *cob* no.: H2 (MF370868), H3 (MF370869), H4 (MF370870) and H5 (MF370871)
Li et al. (2018)	China	Vole (*Neodon*/*Microtus fuscus*)	Sequencing	Haplogroup C1 (JZ03, JZ05, JZ06, JZ07, JZ08, JZ09, JZ10, JZ12), Haplogroup C2 (JZ01, JZ02, JZ04,JZ11, JZ13)	*nad5*, *atp6*, *cox1*, *nad1*	*nad5* (1,914), *atp6* (808), *cox1* (1,801), *nad1* (1,126)	MH259779, MH259780, MH259781, MH259782, MH259783, MH259784, MH259785, MH259786, MH259787, MH259775, MH259776, MH259777, MH259778, MH259764, MH259765, MH259766, MH259767, MH259768, MH259769, MH259770, MH259771, MH259772, MH259773, MH259774
Heidari et al. (2019)	Iran	Red fox (*Vulpes vulpes*), golden jackals (*Canis aureus*)	Sequencing	Haplotypes (E.mKh2, E.mKh4, E.mKh11, E.mKh20, E.mKh22, E.mKh30, E.mKh41, E.mKh47, E.mKh55, E.mKh81	*cox1*, *nad1*	*cox1* (450 of 1,608), *nad1* (500 of 894)	KT318127, KT033486, KT318128, KX186692, KX186693, KX186694, KX186695, KX186696, KX186697, KX186698, KT318129, KT033489, KT318130, KX186699, KX186700, KX186701, KX186702, KX186703, KX186704, KX186705
Knapp et al. (2019)	Sweden, Denmark	Red fox (*Vulpes vulpes*)	Microsatellite	P1 (= G06/G07), P2 (= G05/G07), P3 (= G07), P4 (= G23/G27/G28), P5 (= G19), P6 (= G20/G21)	EmsB	EmsB (209–241)	–
Alvarez Rojas et al. (2020)	Kyrgyzstan	Human (*Homo sapiens*), domestic dog (*Canis lupus familiaris*)	Sequencing	A2, A11-A26	*cob*, *cox1*, *nad2*	*cob* (1,068 of 1,068), *cox1* (1,608 of 1,608), *nad2* (882 of 882)	MN829497-MN829544
Jarošová et al. (2020)	Slovakia	Wolf (*Canis lupus*), domestic dog (*Canis lupus familiaris*)	Sequencing	4 haplotypes	*nad1*, *rrnS*	*nad1* (195 of 894), *rrnS* (149)	MN796073, MN796075, MN796077, MN809175-MN809180, MN813494-MN813498, MH507069, MH744552, MH744553
Knapp et al. (2020)	Belgium, France, Germany, Switzerland	Human (*Homo sapiens*)	Microsatellite	P1 to P9	EmsB	EmsB (209–241)	–
Herzig et al. (2021)	Germany	Red fox (*Vulpes vulpes*)	Sequencing and microsatellite	EmsB (D, G, H)	EmsB, *cob*, *cox1*, *nad1*	EmsB (209–241), *nad1* (379 of 894), *cox1* (785 of 1,608), *atp6* (516 of 516)	–
Knapp et al. (2021a)	France	Red fox (*Vulpes vulpes*), domestic dog *(Canis lupus familiaris*), domestic cat (*Felis catus*)	Microsatellite	Group 1 (G1) - Group 3 (G3)	EmsB	EmsB (209–241)	–
Knapp et al. (2021b)	Switzerland	Domestic pig (*Sus scrofa domesticus*)	Microsatellite	P1 - P12	EmsB	EmsB (209–241)	–
Santa et al. (2021)	Canada	Coyote (*Canis latrans*), red fox (*Vulpes vulpes*)	Sequencing	Haplotypes (*Em ECA*, *Em EAB*, *Em ESK2*, *Em BC1*, *Em N2*, *Em ESK*)	*cob*, *cox1*, *nad1*	*cob* (387 of 1,068), *cox1* (814 of 1,608), *nad1* (344 of 894)	MW591189, MW591188
Shang et al. (2021)	China	Domestic dog (*Canis lupus familiaris*), human (*Homo sapiens*), vole	Sequencing and microsatellite	EmHa1 (*cox1*) - EmHa4 (*cox1*), EmsB (P1 - P7)	*cox1*, EmsB	*cox1* (758 of 1,608), EmsB (209–241)	MZ026364, MZ026301 - MZ026309, MZ026341 - MZ026353, MZ026310 - MZ026322, MZ026363, MZ026323 - MZ026340, MZ026354 - MZ026362
Umhang et al. (2021b)	Poland	Red fox (*Vulpes vulpes*)	Sequencing and microsatellite	*cox1*: European (POL_B, POL_A), Asian (POL_E); EmsB: APol1 (G01; Asian), APol3 (P01; Asian), APol2 (NA; Asian), EPol26 (P19; European), EPol22 (P17; European), EPol34 (P03; European), EPol31 (P24; European)	*cox1*, EmsB	*cox1* (1,608 of 1,608), EmsB (209–241)	MW255891 - MW255894, KY205685, MW255896 - MW255898, KY205677, MW255900, MW255901, KY205685, MW255903 - MW255905, KY205685, MW255907, KY205677, MW255909 - MW255911, KY205685
Umhang et al. (2021a)	France	Vole (*Arvicola terrestris*)	Microsatellite	P22	EmsB	EmsB (209–241)	–
Uakhit et al. (2022)	Kazakhstan	Red Fox (*Vulpes vulpes*)	Sequencing	Hp1, Hp2, Hp3	*cox1*, *nad1*	*cox1* (330 of 1,608), *nad1* (576 or 594 of 894)	OM640355, OM640356, OM471710
Martini et al. (2022)	Luxembourg	Muskrat (*Ondatra zibethicus*)	Sequencing	European (E2, E4, E5)	*cox1*	*cox1* (300-1,590 of 1,608)	–
Polish et al. (2022)	United States	Red fox (*Vulpes vulpes*), human (*Homo sapiens*)	Sequencing	European (E5)	*cox1*, *nad1*, *cob*	*cox1* (?), *nad1* (395 of 894), *cob* (209-902 of 1,068)	–
Santa et al. (2023)	Canada	Red fox (*Vulpes vulpes*), coyote (*Canis latrans*)	Sequencing	ECA, EAB, ESK, ESK2, BC1, N2; EmsB: P1 - PP16	EmsB, *cox1*, *nad2*, *cob*	EmsB (209–241), *nad2* (882 of 882), *cox1* (1,608 of 1,608), *cob* (1,068 of 1,068)	–
Bohard et al. (2023)	France, Luxembourg	Human (*Homo sapiens*)	Sequencing	Europe (Em1-Em13), Asia (EmAsia), EmsB: P4, P8, P9, near 7PRC-rc	Mitogenome, EmsB	mtDNA (13,738 of 13,738), EmsB (209–241)	OQ599939 - OQ599968
Sacheli et al. (2023)	Belgium	Human (*Homo sapiens*)	Microsatellite	P1, P4, P8, P9	EmsB	EmsB (209–241)	–
Karamon et al. (2023)	Poland	Domestic pig (*Sus scrofa domesticus*)	Sequencing	European (EmPL cox A, EmPL cox B, EmPL cox E, EmPL cox F, EmPL cox G, EmPL cox B2 and EmPL cox G2, EmPL nad A2, EmPL nad B2, EmPL nad C2, EmPL nad D2); Asian-like (cox1 (EmPL cox E) and nad2 (Em PL nad B))	*cox1*, *nad2*	*nad2* (882 of 882), *cox1* (1,608 of 1,608)	OQ874673–OQ874679 (*cox1*) and OQ884981–OQ884984 (*nad2*)
Alshammari et al. (2024)	United States, China, Poland, Slovakia, Russia, Austria, France, Kyrgyzstan, Canada, Switzerland, Japan, Hungary, Mongolia, Iran, Sweden, Turkey, Germany, Estonia, Slovenia, Kazakhstan	–	Sequencing	*cox1* (Hap01-20), *nad1* (Hap01-13)	*cox1*, *nad1*	*cox1* (497 of 1,608), *nad1* (285 of 894)	–
Antolová et al. (2024)	Slovakia	Human (*Homo sapiens*)	Sequencing	Haplotypes (E1/E2, E4, E5, E5^a^)	*cox1*, *nad1, cob*, *nad2*	*cox1* (789 of 1,608), *nad1* (395 of 894), *cob* (603 of 1,068), *nad2* (882 of 882)	OP277487-OP277506, OP277507 –OP277525, OP225398, OP225402, OP225448, OP225555, OP225644, OP225830 and OP225945-54, MW326786-7, MW343787-9, MW357715, MW366778-79, MW384819-20 and OP356581-OP356591
Conlon et al. (2024)	United States	Coyote (*Canis latrans*), red fox (*Vulpes vulpes*), gray fox (*Urocyon cinereoargenteus*)	Sequencing and microsatellite	Haplotype G8; EmsB: All New York variants fall within the European clusters	*nad2*, *cob*, EmsB	*nad2* (527 of 882), *cob* (521 of 1,068), EmsB (209–241)	OQ606770–OQ606772, OP596325–OP596330, PQ114719–PQ114730
Hifumi et al. (2024)	Japan, Canada	Hokkaido native pony, light breed, pony, heavy horses	Sequencing	Asian haplogroup, European haplogroup	*cob*	*cob* (694 of 1,068)	LC764417 - LC764425
Lallemand et al. (2024)	France, United States, Alaska, Armenia, Belgium, Canada, China, Germany, Japan, Luxembourg, Norway (Svalbard), Poland, Russia, Sweden, Switzerland	Human (*Homo sapiens*), tundra vole (*Microtus oeconomus*), rodent, domestic dog (*Canis lupus familiaris*), muskrat (*Ondatra zibethicus*), monkey, grey-sided Vole (*Clethrionomys rufocanus*), red fox (*Vulpes vulpes*), sibling vole (*Microtus levis*), Mongolian gerbil (*Meriones unguiculatus*), narrow-headed vole (*Microtus gregalis*), lake Baikal mountain vole (*Alticola olchonensis*), vervet monkey (*Chlorocebus pygerythrus*)	Sequencing	Haplogroups (HG1, HG2, HG3a, HG3b, HG3c, HG4)	Mitogenome	mtDNA (13,735 to 13,740 of 13,738)	OR911371-OR911453
Santoro et al. (2024)	Austria, Belgium, Croatia, Czech Republic, Denmark, Estonia, France, Germany, Hungary, Italy, Latvia, Lithuania, Luxembourg, Norway (Svalbard archipelago), Poland, Slovakia, Switzerland	Red fox (*Vulpes vulpes*), Japanese macaque (*Macaca fuscata*), human (*Homo sapiens*), raccoon dog (*Nyctereutes procyonoides*), Eurasian beaver (*Castor fiber*), mouse, common vole (*Microtus arvalis*), wolf (*Canis lupus*), muskrat (*Ondatra zibethicus*), arctic fox (*Vulpes lagopus*), golden jackal (*Canis aureus*), western gorilla (*Gorilla gorilla*), crab-eating macaque (*Macaca fascicularis*), domestic dog (*Canis lupus familiaris*), wild boar (*Sus scrofa*), domestic pig (*Sus scrofa domesticus*), ring-tailed lemur (*Lemur catta*)	Sequencing	Haplotype H1 to H43	*cox1*, *nad1*, *atp6*, *cob*, *nad2*	*cox1* (1,608 of 1,608), *nad1* (588 of 894), *atp6* (516 of 516), *cob* (663 of 1,068), *nad2* (882 of 882)	See Supplementary Table S3 Santoro et al. (2024)
Wang et al. (2024)	China	Narrow-headed vole (*Microtus gregalis*), Tien Shan vole (*Microtus ilaeus*), northern mole vole (*Ellobius talpinus*)	Sequencing	Haplotype Hap_1 - Hap_21	*nad1*	*nad1* (510 of 894)	–
Guo et al. (2024)	China	Human (*Homo sapiens*), small rodent, domestic dog (*Canis lupus familiaris*)	Sequencing	Haplotype Hap1 - Hap27	*cox1*, *cob*, *nad2*	*cox1* (1,608 of 1,608), *cob* (1,068 of 1,068), *nad2* (882 of 882)	see **Table 1** Guo et al. (2024)
Guo et al. (2025)	United States, Japan, China	mouse models	Sequencing	Asian/European geographical clade (EM-XJ, EM-JP, EM-NX), North American clade (EM-AK)	Mitogenome	mtDNA (13,738)	OP628492–OP628495
Cafiero et al. (2025)	Italy	Wolf (*Canis lupus*), red fox (*Vulpes, vulpes*)	Sequencing	4 Haplotypes	*cox1*	*cox1* (355 of 1,608)	PQ479227, PQ479228, PQ479229, PQ479230

The information and designations of the haplotypes/genotypes/profiles correspond to those in the respective paper. This table does not claim to be exhaustive of all papers ever written on the subject. The abbreviations for the genes/markers stand for: DNA = Deoxyribonucleic Acid; RNA = Ribonucleic Acid; pAL 1 = the name of a recombinant plasmid; *cox1* = gene name of Cytochrome c oxidase subunit I; *cob* = gene name of Cytochrome b; *atp6* = gene name of ATP synthase subunit 6; *nad1* = gene name of NADH dehydrogenase subunit 1; *nad2* = gene name of NADH dehydrogenase subunit 2; *nad5* = gene name of NADH dehydrogenase subunit 5; *rrnS* = gene name of 12S ribosomal RNA (small subunit); mitogenome = complete mitochondrial DNA (mtDNA); *ef1a* = gene name of nuclear (gDNA) elongation factor-1 alpha; *elp* = gene name of nuclear ezrin-radixin-moesin-like protein; *cal* = gene name of nuclear calreticulin; *th* = gene name of nuclear thioredoxin peroxidase; ActII = Actin II (non-coding intron region); Hbx2 = Homeobox protein 2 (non-coding intron region); AgB/1 = nuclear antigen B subunit 1 (coding region); ITS1 = first internal transcribed spacers; ITS2 = second internal transcribed spacers; (CAC)_5_ = oligonucleotide probe; EmsB = tandem repeated multi-loci microsatellite; EmsJ, EmsK, and NAK1 = single-locus microsatellites; U1 snRNA = U1 small nuclear RNA; EMms1, EMms2 = two microsatellite loci; bp = base pairs.

The genetic diversity of *E. multilocularis* has been studied since the early 1990s. Initially, there were three possible methods for analysing the DNA of *E. multilocularis*: PCR, Sanger Sequencing and comparative analysis of mitochondrial genes (Maxam and Gilbert, 1977; Sanger et al., 1977, Sanger et al., 1980; Saiki et al., 1985; Avise et al., 1987; Tajima, 1989; Bowles et al., 1992; Excoffier et al., 1992; Bowles and McManus, 1993a). The PCR method made it possible to amplify specific regions of the genome for the first time (Gottstein and Mowatt, 1991; Bretagne et al., 1993), Sanger Sequencing allowed the determination of the nucleotide sequence (Bowles et al., 1992), and comparative analysis helped to clarify phylogenetic relationships and population structure (Bowles et al., 1995). While initially only short fragments of individual genes could be sequenced (Bowles and McManus, 1993b), the determined sequences became longer and longer over the course of the following decades, until several entire genes (Herzig et al., 2021) (see also **Table 1**) and finally the entire mitogenome DNA sequence served as the basis for the respective studies (Nakao et al., 2002; Bohard et al., 2023; Lallemand et al., 2024; Guo et al., 2025; Rachel et al., 2025).

Parts of genomic DNA (gDNA) were also used as markers. These were mainly microsatellite markers (Bretagne et al., 1996; Nakao et al., 2003; Bart et al., 2006; Knapp et al., 2007). One microsatellite marker, named EmsB, was used particularly frequently (e.g. Knapp et al., 2009; Casulli et al., 2010; Umhang et al., 2017; Sacheli et al., 2023). However, it became apparent that samples from different regions are always required for the creation of EmsB dendrograms, as the marker and the statistical method applied to analyse the data influence the cluster structure of the dendrogram due to the number of individual variations in the used samples (Knapp et al., 2020; Umhang et al., 2021b). There was also further criticism (Mohammadi and Harandi, 2024) of the EmsB studies, limiting the significance and accuracy of these papers and suggesting that studying the diversity of *E. multilocularis* could be improved by using other genetic methods (e.g. NGS) that cover a broader range of genetic markers.

Older studies often did not use complete genes or applied sequences from multiple different genes or genetic markers, respectively. Moreover, when the sequences of the same gene (e.g. *cox1* or *nad1*) were examined across studies, these often differed in their length, or only the “informative” sections of the gene fragments were included in the analysis (Antolová et al., 2024). Moreover, the nomenclature of the identified genotypes and haplotypes was inconsistent, with some studies even reversing names (Šnábel et al., 2020). These inconsistencies resulted in a situation, where little to no reliable assessment of the genetic diversity of *E. multilocularis* could be derived from the literature of the past decades, as the data are largely incomparable (e.g., mtDNA, microsatellites of gDNA) (**Table 1**).

Given the current critical perspective on EmsB microsatellite analysis, hypotheses derived from this method—such as the mainland-island model proposed by Knapp et al. (2009)—require confirmation through alternative genetic approaches. This is particularly important because other studies employing mitochondrial DNA markers have either documented an east-to-west spread of haplotypes (Nakao et al., 2009) or have been unable to determine a directional pattern definitively, discussing multiple possible scenarios (Karamon et al., 2017). It is important to note, however that the sample sizes studied by Nakao et al. (2009) and Knapp et al. (2009) and the origin of the specimens were different, thus, the conclusions on the circulation of the respective strains in the study regions were not identical. Yet, integrating diverse genetic methods is essential to elucidate the population dynamics and dispersal routes of *E. multilocularis* robustly. Although good results have already been achieved with a few mtDNA genes, it may be preferable to analyse the entire mitogenome for the investigation of genetic diversity as already done by some investigators (Bohard et al., 2023; Lallemand et al., 2024; Guo et al., 2025; Rachel et al., 2025). This approach has the advantage that, once a good marker for the genetic diversity of *E. multilocularis* has been identified, the data from older studies can also be validated and may also be used to examine specific sections of these sequences. Furthermore, a study by Guo et al. (2025) analysed mitogenome sequences from a limited number of *E. multilocularis* isolates and provided preliminary evidence suggesting a potential correlation between mitogenome sequence-based genotypes and the virulence of the respective strains in laboratory mice. However, due to the small sample size, these findings should be interpreted with caution and regarded preliminary or as an initial indication warranting further investigation.

Due to methodological differences outlined above, the genetic diversity of *E. multilocularis* has been reported variably across studies. Early investigations, which analysed only very short mitochondrial DNA segments, estimated the genetic diversity of the parasite to be quite low (Bowles et al., 1992; Bowles and McManus, 1993a; Okamoto et al., 1995). By contrast, analyses of the same limited DNA segments in *E. granulosus* revealed substantially higher genetic diversity, erroneously leading to the conclusion that diversity in *E. multilocularis* is low. Consequently, the findings of earlier studies are of limited value (Bowles et al., 1992; Bowles and McManus, 1993a).

The analysis of the complete mtDNA also shows a larger number of Single Nucleotide Polymorphisms (SNPs), which led to a larger number of haplotypes and thus indicates greater genetic diversity, but also greater intraspecific variation (Bohard et al., 2023; Lallemand et al., 2024; Guo et al., 2025). While an early study (Nakao et al., 2009) showed that the *E. multilocularis* population can be divided on a continental level (with clades for Europe, Asia, and North America), the latest investigations (Bohard et al., 2023; Lallemand et al., 2024; Guo et al., 2025) demonstrate that it is possible to identify regions with specific haplotypes. Bohard et al. (2023) identified 13 different haplotypes in human samples from France. Lallemand et al. (2024) analysed samples from France, Asia, North America and the Arctic. They detected 58 haplotypes, which were grouped into four haplogroups (with three micro-haplogroups). These groups could be assigned to specific regions. Haplogroup HG1 was identified in Alaska, St. Lawrence Island, Yakutia (Russia) and Svalbard (Norway), while HG2 occurred in Asia, North America and Europe. Haplogroup HG3 could be further divided into three micro-haplogroups (HG3a: North America, Europe; HG3b and HG3c: Europe). A fourth haplogroup, HG4, comprised only one isolate from Olkhon Island (Russia). Guo et al. (2025), examined the samples for intraspecific variation and found that the strain from Alaska (EM-AK) produced more protoscolices than the other strains tested and that the EM-AK strain triggered a stronger inflammatory response and liver fibrosis in laboratory mice than the other three strains. This EM-AK strain belongs to the North American clade. The data from the studies, where the complete mitogenome sequences were applied, have shown that the diversity is higher than was assumed a few years ago. If the complete gDNA (created by WGS) is be used in the future, it will probably be possible to increase the knowledge on the genetic diversity within the species *E. multilocularis* even further and it may ultimately become possible to obtain a more detailed resolution of genetic diversity at the local level.

## Genotyping methods

When research into the genetic diversity of *E. multilocularis* began, DNA cloning techniques were used (Vogel et al., 1990). This was followed by analysis using various markers (**Table 1**). Over time, two techniques proved to be suitable and were widely used. These were the examination of mitochondrial markers (fragments of genes, whole genes or the complete mitogenome) by means of sequencing (e.g. Sanger sequencing, NGS) and EmsB microsatellite studied by fragment size analysis.

The EmsB microsatellite marker of *E. multilocularis* is located on chromosome 5 in the genome (gDNA) of the parasite. It is present in 40 copies, which are arranged in tandem and have a (CA)_n_(GA)_m_ pattern (Bart et al., 2006; Valot et al., 2015). The described advantages of the EmsB method are its high discriminatory power, its applicability to different sample types, and its straightforward workflow (Valot et al., 2015; Knapp et al., 2020; Sacheli et al., 2023). The discussed disadvantages are that special equipment is required, interpretation of the results is not easy, the genetic diversity of *E. multilocularis* is underestimated because only a small part of the gDNA is used, and comparability can be difficult, which is at least partially due to the method applied for normalisation and evaluation of raw microsatellite data (Bart et al., 2006; Valot et al., 2015; Knapp et al., 2020).

For reasons of practicality, robustness and cost-effectiveness, some research groups investigating the genetic diversity of *E. multilocularis* tend to employ sequencing techniques—particularly Next-Generation Sequencing (NGS)—and Single Nucleotide Polymorphism (SNP) analysis (Bohard et al., 2023; Lallemand et al., 2024; Mohammadi and Harandi, 2024; Rachel et al., 2025). Different NGS systems have unique characteristics, each with specific advantages and disadvantages (Pedroza Matute and Iyavoo, 2025). Some advantages of these methods are their high discriminatory power, the ability to examine additional loci, while maintaining short amplicon sizes, and, in combination with SNP analysis (low mutation rate), NGS is able to perform more stable ancestry tracking and has a high multiplex capacity (Pedroza Matute and Iyavoo, 2025). Furthermore, more markers can be found and analysed per run (Davey et al., 2011). Due to the fact that the entire genetic sequence is analysed, a more complete genetic picture can be created by recording SNPs, INDELs, etc (Satam et al., 2023, 2024). Disadvantages of NGS can include high costs (although these have fallen significantly in recent years), high complexity of the sequencing devices and the need for expertise in bioinformatics to evaluate the data (Pedroza Matute and Iyavoo, 2025). Furthermore, huge amounts of data require a lot of storage space, usually in the form of servers (Bagger et al., 2024). A further potential limitation of NGS methods lies in the high costs for equipment, which may restrict their use to a limited number of laboratories. However, NGS analysis can nowadays be outsourced to external service laboratories and recent advances for example in nanopore technology (e.g. by Oxford Nanopore Technologies) have introduced affordable devices that promote broader accessibility to NGS technology. Yet, last but not least, the amount and quality of the DNA must be sufficient to perform NGS and obtain valuable results (McNulty et al., 2020).

While NGS methods can be costly and require a great deal of IT knowledge for evaluation (e.g. administration of IT infrastructure, development of required bioinformatic pipelines, and management of these via respective workflows), they are well suited to analyse genetic diversity, as they allow the complete parasite genome to be sequenced, which can increase the detection of variable sites. Furthermore, read data from full-length gene sequences—in comparison with gene fragments—permit more robust harmonisation and cross-study evaluation, thus facilitating integrated analysis of genetic polymorphisms and population diversity in *E. multilocularis*. Furthermore, by analysing SNPs, they could potentially identify strains that are particularly virulent in some species (Guo et al., 2025), which may be relevant for the treatment and prognosis of patients with alveolar echinococcosis (AE) if applicable to humans.

## Epidemiological impact of genetic diversity

Single Nucleotide Polymorphisms (SNPs) may alter protein structure, thereby affecting binding capacity, transmission dynamics, parasite density within the host, and the virulence of *E. multilocularis* (Wen et al., 2019). Genetic polymorphisms usually occurs due to evolutionary pressure as a result of a change in the system (new host, altered environmental conditions due to, for example, the host migrating to another region) (Kim and Shaw, 2021). This can have implications for the host. For example, a different haplotype seems to have a different virulence, or even the morphology of the parasite may be slightly different (Guo et al., 2025). Such changes could, for example, result in altered drug efficacy or modified metabolic processing by the parasite, potentially impacting treatment outcomes and consequently affecting the survival probability of patients with alveolar echinococcosis (AE). Furthermore, intraspecific ‘specialisation’ of certain haplotypes for certain hosts may occur. In North America, certain haplotypes were found that prefer coyotes as hosts, as well as haplotypes that are particularly virulent (Nakao et al., 2009; Alvarez Rojas et al., 2020; Guo et al., 2025). This results in different distribution patterns of *E. multilocularis* in different host populations. These genetic differences may thus be important for the surveillance of *E. multilocularis*, as they could be used to identify and control sources of infection and hotspots in the environment.

## Future research and challenges

Despite many years of research, our knowledge of the genetic diversity of *E. multilocularis* is still limited. However, new methods and improved sequencing techniques (e.g. further development of 3^rd^ generation long read NGS platform technology) may make this research more efficient and less costly in the future (Santa et al., 2021; Satam et al., 2023; Espinosa et al., 2024; Satam et al., 2024; Rachel et al., 2025). The adoption of a standardised nomenclature for SNPs—such as that established for the human genome (Hart et al., 2024; Phan et al., 2025)—would be desirable to facilitate more effective, harmonised comparison and rapid evaluation of genotype and haplotype data for *E. multilocularis* in future studies. It would also be beneficial if more working groups aimed to obtain complete mitogenome sequences from their samples. This could make it possible to combine data obtained with newly discovered marker regions and information established with older sequence data and to analyse them together. The use of complete gDNA may also be a future goal to expand genetic diversity. This would make it possible to identify potential chains of infection at the local level and take measures to reduce the number of *E. multilocularis* infections, especially in endemic areas, thereby protecting humans and animals.

## Conclusion

It was only in the last two years, that research methods have matured to the point where good data could be collected for the evaluation of genetic diversity of *E. multilocularis*, thanks to the use of NGS and the examination of the mitogenome using SNPs. Furthermore, older findings have also been confirmed in the new studies, such as the discovery that there are three clades of *E. multilocularis* (Europe, Asia, North America) (Nakao et al., 2009; Spotin et al., 2018; Šnábel et al., 2020; Guo et al., 2025). The virulence of individual strains to particular host species has also been determined (Guo et al., 2025). It seems now important to improve cooperation within the *E. multilocularis* research community, to establish a uniform nomenclature for the naming of haplotypes/genotypes and SNPs and to harmonise the bioinformatic analysis of *E. multilocularis* genome sequences so that studying the genetic diversity of this parasite can be further improved.

## References

[B1] Abulizi A. Wen H. Zhang C. Li L. Ran B. Jiang T. . (2018). Sequence analysis of mitochondrial cytochrome c oxidase 1 and cytochrome b genes of echinococcus multilocularis from human patients. Int. J. Clin. Exp. Pathol. 11, 795–801., PMID: 31938167 PMC6958033

[B2] Alshammari A. Subhani M. Wakid M. Alkhaldi A. Hussain S. Malik M. . (2024). Genetic diversity and population structure of Echinococcus multilocularis: An in-silico global analysis. J. Advanced Veterinary Anim. Res. 11, 264–274. doi: 10.5455/javar.2024.k772, PMID: 39101071 PMC11296166

[B3] Alvarez Rojas C. Kronenberg P. Aitbaev S. Omorov R. Abdykerimov K. Paternoster G. . (2020). Genetic diversity of Echinococcus multilocularis and Echinococcus granulosus sensu lato in Kyrgyzstan: The A2 haplotype of E. multilocularis is the predominant variant infecting humans. PLoS Negl. Trop. Dis. 14, e0008242–e0008242. doi: 10.1371/journal.pntd.0008242, PMID: 32401754 PMC7219741

[B4] Antolová D. Šnábel V. Jarošová J. Cavallero S. D’Amelio S. Syrota Y. . (2024). Human alveolar echinococcosis in Slovakia: Epidemiology and genetic diversity of Echinococcus multilocularis 2000-2023. PLoS Negl. Trop. Dis. 18, e0011876. doi: 10.1371/journal.pntd.0011876, PMID: 38198452 PMC10805277

[B5] Autier B. Gottstein B. Millon L. Ramharter M. Gruener B. Bresson-Hadni S. . (2022). “ Alveolar echinococcosis in immunocompromised hosts,” in Clinical microbiology and infection: the official publication of the European Society of Clinical Microbiology and Infectious Diseases (United Kingdom: Elsevier B.V.). doi: 10.1016/j.cmi.2022.12.010, PMID: 36528295

[B6] Avise J. Arnold J. Ball R. Bermingham E. Lamb T. Neigel J. . (1987). Intraspecific phylogeography: the mitochondrial DNA bridge between population genetics and systematics. Annu. Rev. Ecol. Systematics 18, 489–522. doi: 10.1146/annurev.es.18.110187.002421

[B7] Bagger F. Borgwardt L. Jespersen A. Hansen A. Bertelsen B. Kodama M. . (2024). Whole genome sequencing in clinical practice. BMC Med. Genomics 17, 39. doi: 10.1186/s12920-024-01795-w, PMID: 38287327 PMC10823711

[B8] Bagrade G. Šnábel V. Romig T. Ozoliņš J. Hüttner M. Miterpáková M. . (2008). Echinococcus multilocularis is a frequent parasite of red foxes (Vulpes vulpes) in Latvia. Helminthologia 45, 157–161. doi: 10.2478/s11687-008-0032-1

[B9] Bart J.-M. Breyer I. Gottstein B. Romig T. Piarroux R. (2003). Development of molecular tools to explore genetic diversity in Echinococcus multilocularis. Helminthologia 117–121.

[B10] Bart J. M. Knapp J. Gottstein B. El-Garch F. Giraudoux P. Glowatzki M. L. . (2006). EmsB, a tandem repeated multi-loci microsatellite, new tool to investigate the genetic diversity of Echinococcus multilocularis. Infection Genet. Evol. 6, 390–400. doi: 10.1016/j.meegid.2006.01.006, PMID: 16504596

[B11] Bohard L. Lallemand S. Borne R. Courquet S. Bresson-Hadni S. Richou C. . (2023). Complete mitochondrial exploration of Echinococcus multilocularis from French alveolar echinococcosis patients. Int. J. Parasitol. 53, 555–564. doi: 10.1016/j.ijpara.2023.03.006, PMID: 37148987

[B12] Bowles J. Blair D. McManus D. (1992). Genetic variants within the genus Echinococcus identified by mitochondrial DNA sequencing. Mol. Biochem. Parasitol. 54, 165–173. doi: 10.1016/0166-6851(92)90109-W, PMID: 1435857

[B13] Bowles J. Blair D. McManus D. (1995). A molecular phylogeny of the genus Echinococcus. Parasitology 110, 317–328. doi: 10.1017/S0031182000080902, PMID: 7724239

[B14] Bowles J. McManus D. (1993a). Molecular variation in echinococcus. Acta Tropica 53, 291–305. doi: 10.1016/0001-706X(93)90035-A, PMID: 8100676

[B15] Bowles J. McManus D. (1993b). NADH dehydrogenase 1 gene sequences compared for species and strains of the genus Echinococcus. Int. J. Parasitol. 23, 969–972. doi: 10.1016/0020-7519(93)90065-7, PMID: 8106191

[B16] Bretagne S. Assouline B. Vidaud D. Houin R. Vidaud M. (1996). Echinococcus multilocularis: microsatellite polymorphism in U1 snRNA genes. Exp. Parasitol. 82, 324–328. doi: 10.1006/expr.1996.0040, PMID: 8631384

[B17] Bretagne S. Guillou J. Morand M. Houin R. (1993). Detection of Echinococcus multilocularis DNA in fox faeces using DNA amplification. Parasitology 106, 193–199. doi: 10.1017/S0031182000074990, PMID: 8446472

[B18] Cafiero S. A. Petroni L. Natucci L. Tomassini O. Romig T. Wassermann M. . (2025). New evidence from the northern Apennines, Italy, suggests a southward expansion of Echinococcus multilocularis range in Europe. Sci. Rep. 15, 7353. doi: 10.1038/s41598-025-91596-7, PMID: 40025062 PMC11873164

[B19] Casulli A. Bart J. Knapp J. La Rosa G. Dusher G. Gottstein B. . (2009). Multi-locus microsatellite analysis supports the hypothesis of an autochthonous focus of Echinococcus multilocularis in northern Italy. Int. J. Parasitol. 39, 837–842. doi: 10.1016/j.ijpara.2008.12.001, PMID: 19150351

[B20] Casulli A. Széll Z. Pozio E. Sréter T. (2010). Spatial distribution and genetic diversity of Echinococcus multilocularis in Hungary. Veterinary Parasitol. 174, 241–246. doi: 10.1016/j.vetpar.2010.08.023, PMID: 20880633

[B21] Casulli A. Tamarozzi F. (2021). Tracing the source of infection of cystic and alveolar echinococcosis, neglected parasitic infections with long latency: The shaky road of “evidence” gathering. PLoS Negl. Trop. Dis. 15, e0009009. doi: 10.1371/journal.pntd.0009009, PMID: 33476336 PMC7819598

[B22] Centers for Disease Control and Prevention (2019). Echinococcosis. Available online at: https://www.cdc.gov/dpdx/echinococcosis/ (Accessed July 15, 2019).

[B23] Conlon C. Brigandi J. Frair J. Michaud-LeBlanc C. Schuler K. Lejeune M. . (2024). Echinococcus multilocularis in new york wildlife: distribution and genetic diversity of an emerging pathogen. para 110, 697–708. doi: 10.1645/24-54, PMID: 39701161

[B24] Conraths F. Deplazes P. (2015). Echinococcus multilocularis: Epidemiology, surveillance and state-of-the-art diagnostics from a veterinary public health perspective. Veterinary Parasitol. 213, 149–161. doi: 10.1016/j.vetpar.2015.07.027, PMID: 26298509

[B25] Conraths F. Maksimov P. (2020). Epidemiology of Echinococcus multilocularis infections: A review of the present knowledge and of the situation in Germany. Berliner und Münchener Tierärztliche Wochenschrift 133. doi: 10.2376/0005-9366-2020-5

[B26] Conraths F. Probst C. Possenti A. Boufana B. Saulle R. La Torre G. . (2017). Potential risk factors associated with human alveolar echinococcosis: Systematic review and meta-analysis. PLoS Negl. Trop. Dis. 11, 1–15. doi: 10.1371/journal.pntd.0005801, PMID: 28715408 PMC5531747

[B27] Davey J. Hohenlohe P. Etter P. Boone J. Catchen J. Blaxter M. (2011). Genome-wide genetic marker discovery and genotyping using next-generation sequencing. Nat. Rev. Genet. 12, 499–510. doi: 10.1038/nrg3012, PMID: 21681211

[B28] Deplazes P. Rinaldi L. Alvarez Rojas C. Torgerson P. Harandi M. Romig T. . (2017). Global distribution of alveolar and cystic echinococcosis. Adv. Parasitol. 95, 315–493. doi: 10.1016/bs.apar.2016.11.001, PMID: 28131365

[B29] Dyachenko V. Pantchev N. Gawlowska S. Vrhovec M. G. Bauer C. (2008). Echinococcus multilocularis infections in domestic dogs and cats from Germany and other European countries. Veterinary Parasitol. 157, 244–253. doi: 10.1016/j.vetpar.2008.07.030, PMID: 18819752

[B30] Espinosa E. Bautista R. Larrosa R. Plata O. (2024). Advancements in long-read genome sequencing technologies and algorithms. Genomics 116, 110842. doi: 10.1016/j.ygeno.2024.110842, PMID: 38608738

[B31] Excoffier L. Smouse P. Quattro J. (1992). Analysis of molecular variance inferred from metric distances among DNA haplotypes: application to human mitochondrial DNA restriction data. Genetics 131, 479–491. doi: 10.1093/genetics/131.2.479, PMID: 1644282 PMC1205020

[B32] Furtado Jost R. Müller N. Marreros N. Moré G. Antoine L. Basso W. . (2023). What is the role of Swiss domestic cats in environmental contamination with Echinococcus multilocularis eggs? Parasit Vectors 16, 353. doi: 10.1186/s13071-023-05983-y, PMID: 37807080 PMC10561489

[B33] Gesy K. Jenkins E. (2015). Introduced and native haplotypes of echinococcus multilocularis in wildlife in Saskatchewan, Canada. J. Wildl Dis. 51, 743–748. doi: 10.7589/2014-08-214, PMID: 26020284

[B34] Gesy K. Schurer J. Massolo A. Liccioli S. Elkin B. Alisauskas R. . (2014). Unexpected diversity of the cestode Echinococcus multilocularis in wildlife in Canada. Int. J. Parasitol. Parasites Wildl 3, 81–87. doi: 10.1016/j.ijppaw.2014.03.002, PMID: 25161905 PMC4142260

[B35] Gottstein B. Mowatt M. (1991). Sequencing and characterization of an Echinococcus multilocularis DNA probe and its use in the polymerase chain reaction. Mol. Biochem. Parasitol. 44, 183–193. doi: 10.1016/0166-6851(91)90004-P, PMID: 2052020

[B36] Guo B. Guo G. Qi W. Aizezi M. Wu C. Tian M. . (2025). The genetic variation of mitochondrial sequences and pathological differences of Echinococcus multilocularis strains from different continents. Microbiol. Spectr. 13, e01318–e01324. doi: 10.1128/spectrum.01318-24, PMID: 39950816 PMC11960119

[B37] Guo B. Wu C. Wang J. Wang W. Ren B. Yuan A. . (2024). The A2 haplotype of Echinococcus multilocularis is the predominant variant infecting humans and dogs in Yili Prefecture, Xinjiang. Infect. Genet. Evol. 119, 105581. doi: 10.1016/j.meegid.2024.105581, PMID: 38432594

[B38] Haag K. Zaha A. Araújo A. Gottstein B. (1997). Reduced genetic variability within coding and non-coding regions of the Echinococcus multilocularis genome. Parasitology 115, 521–529. doi: 10.1017/s0031182097001649, PMID: 9368903

[B39] Hart R. Fokkema I. DiStefano M. Hastings R. Laros J. Taylor R. . (2024). HGVS Nomenclature 2024: improvements to community engagement, usability, and computability. Genome Med. 16, 149. doi: 10.1186/s13073-024-01421-5, PMID: 39702242 PMC11660784

[B40] Hegglin D. Deplazes P. (2013). Control of Echinococcus multilocularis: strategies, feasibility and cost-benefit analyses. Int. J. Parasitol. 43, 327–337. doi: 10.1016/j.ijpara.2012.11.013, PMID: 23384814

[B41] Heidari Z. Sharbatkhori M. Mobedi I. Mirhendi S. Nikmanesh B. Sharifdini M. . (2019). Echinococcus multilocularis and Echinococcus granulosus in canines in North-Khorasan Province, northeastern Iran, identified using morphology and genetic characterization of mitochondrial DNA. Parasit Vectors 12, 606. doi: 10.1186/s13071-019-3859-z, PMID: 31881913 PMC6935109

[B42] Herzig M. (2019). Molekulare Typisierung von Echinococcus multilocularis-Isolaten aus Deutschland. Friedrich-Schiller-Universität, Fakultät für Biowissenschaften, Jena.

[B43] Herzig M. Maksimov P. Staubach C. Romig T. Knapp J. Gottstein B. . (2021). Red foxes harbor two genetically distinct, spatially separated Echinococcus multilocularis clusters in Brandenburg, Germany. Parasit Vectors 14, 535. doi: 10.1186/s13071-021-05038-0, PMID: 34649615 PMC8518320

[B44] Hifumi T. Tanaka T. Suzu I. Sato M. Akioka K. Fujimata C. . (2024). Molecular phylogenetic analysis of Echinococcus multilocularis from horses raised in Canada or Japan, using mitochondrial cytochrome b gene-targeted PCR. Food Waterborne Parasitol. 34, e00219. doi: 10.1016/j.fawpar.2024.e00219, PMID: 38298421 PMC10827676

[B45] Jarošová J. Antolová D. Šnábel V. Guimarães N. Štofík J. Urban P. . (2020). The fox tapeworm, Echinococcus multilocularis, in grey wolves and dogs in Slovakia: epidemiology and genetic analysis. J. Helminthology 94, e168. doi: 10.1017/S0022149X20000528, PMID: 32624011

[B46] Kamiya M. Ooi H. Oku Y. Yagi K. Ohbayashi M. (1985). Growth and development of Echinococcus multilocularis in experimentally infected cats. Japanese J. veterinary Res. 33, 135–140. doi: 10.14943/jjvr.33.3-4.135 4087637

[B47] Kapel C. Torgerson P. Thompson R. Deplazes P. (2006). Reproductive potential of Echinococcus multilocularis in experimentally infected foxes, dogs, raccoon dogs and cats. Int. J. Parasitol. 36, 79–86. doi: 10.1016/j.ijpara.2005.08.012, PMID: 16199043

[B48] Karamon J. Samorek-Pieróg M. Bilska-Zając E. Korpysa-Dzirba W. Sroka J. Bełcik A. . (2023). Echinococcus multilocularis genetic diversity based on isolates from pigs confirmed the characteristic haplotype distribution and the presence of the Asian-like haplotype in Central Europe. J. veterinary Res. 67, 567–574. doi: 10.2478/jvetres-2023-0056, PMID: 38130462 PMC10730556

[B49] Karamon J. Sroka J. Dąbrowska J. Bilska-Zając E. Zdybel J. Kochanowski M. . (2019). First report of Echinococcus multilocularis in cats in Poland: A monitoring study in cats and dogs from a rural area and animal shelter in a highly endemic region. Parasites Vectors 12, 1–8. doi: 10.1186/s13071-019-3573-x, PMID: 31234884 PMC6591820

[B50] Karamon J. Stojecki K. Samorek-Pierog M. Bilska-Zajac E. Rozycki M. Chmurzynska E. . (2017). Genetic diversity of Echinococcus multilocularis in red foxes in Poland: the first report of a haplotype of probable Asian origin. Folia Parasitologica 64, 1–6. doi: 10.14411/fp.2017.007, PMID: 28360380

[B51] Kim D. Shaw A. (2021). Migration and tolerance shape host behaviour and response to parasite infection. J. Anim. Ecol. 90, 2315–2324. doi: 10.1111/1365-2656.13539, PMID: 34014562

[B52] Knapp J. Bart J.-M. Giraudoux P. Glowatzki M.-L. Breyer I. Raoul F. . (2009). Genetic diversity of the cestode Echinococcus multilocularis in red foxes at a continental scale in Europe. PLoS Negl. Trop. Dis. 3, e452–e452. doi: 10.1371/journal.pntd.0000452, PMID: 19513103 PMC2685985

[B53] Knapp J. Bart J. Glowatzki M. Ito A. Gerard S. Maillard S. . (2007). Assessment of use of microsatellite polymorphism analysis for improving spatial distribution tracking of echinococcus multilocularis. J. Clin. Microbiol. 45, 2943–2950. doi: 10.1128/JCM.02107-06, PMID: 17634311 PMC2045259

[B54] Knapp J. Combes B. Umhang G. Aknouche S. Millon L. (2016). Could the domestic cat play a significant role in the transmission of Echinococcus multilocularis? A study based on qPCR analysis of cat feces in a rural area in France. Parasite 23, 42. doi: 10.1051/parasite/2016052, PMID: 27739398 PMC5782850

[B55] Knapp J. Da Silva A. Courquet S. Millon L. (2021a). Assessment of the genetic diversity of echinococcus multilocularis from copro-isolated eggs. Pathogens 10, 1296. doi: 10.3390/pathogens10101296, PMID: 34684245 PMC8541330

[B56] Knapp J. Gottstein B. Bretagne S. Bart J.-M. Umhang G. Richou C. . (2020). Genotyping echinococcus multilocularis in human alveolar echinococcosis patients: an emsB microsatellite analysis. Pathogens 9, 1–21. doi: 10.3390/pathogens9040282, PMID: 32295095 PMC7238142

[B57] Knapp J. Gottstein B. Saarma U. Millon L. (2015). Taxonomy, phylogeny and molecular epidemiology of Echinococcus multilocularis: From fundamental knowledge to health ecology. Veterinary Parasitol. 213, 85–91. doi: 10.1016/j.vetpar.2015.07.030, PMID: 26260408

[B58] Knapp J. Guislain M. Bart J. Raoul F. Gottstein B. Giraudoux P. . (2008). Genetic diversity of Echinococcus multilocularis on a local scale. Infection Genet. Evol. 8, 367–373. doi: 10.1016/j.meegid.2008.02.010, PMID: 18406214

[B59] Knapp J. Meyer A. Courquet S. Millon L. Raoul F. Gottstein B. . (2021b). Echinococcus multilocularis genetic diversity in Swiss domestic pigs assessed by EmsB microsatellite analyzes. Veterinary Parasitol. 293, 109429. doi: 10.1016/j.vetpar.2021.109429, PMID: 33895467

[B60] Knapp J. Staebler S. Bart J. Stien A. Yoccoz N. Drögemüller C. . (2012). Echinococcus multilocularis in Svalbard, Norway: microsatellite genotyping to investigate the origin of a highly focal contamination. Infect. Genet. Evol. 12, 1270–1274. doi: 10.1016/j.meegid.2012.03.008, PMID: 22465539

[B61] Knapp J. Umhang G. Wahlström H. Al-Sabi M. Ågren E. Enemark H. (2019). Genetic diversity of Echinococcus multilocularis in red foxes from two Scandinavian countries: Denmark and Sweden. Food Waterborne Parasitol. 14, e00045. doi: 10.1016/j.fawpar.2019.e00045, PMID: 32095608 PMC7033969

[B62] Konyaev S. Yanagida T. Nakao M. Ingovatova G. Shoykhet Y. Bondarev A. . (2013). Genetic diversity of Echinococcus spp. in Russia. Parasitology 140, 1637–1647. doi: 10.1017/S0031182013001340, PMID: 23985385

[B63] Lallemand S. Oyhenart J. Valot B. Borne R. Bohard L. Umhang G. . (2024). Challenging the phylogenetic relationships among Echinococcus multilocularis isolates from main endemic areas. Int. J. Parasitol. 54, 569–582. doi: 10.1016/j.ijpara.2024.05.004, PMID: 38815855

[B64] Leuckart R. (1863). Die menschlichen Parasiten und die von ihnen herrührenden Krankheiten. Ein Hand- und Lehrbuch für Naturforscher und Aerzte (Leipzig: C.F. Winter’sche Verlagshandlung).

[B65] Li J.-Q. Li L. Fan Y.-L. Fu B.-Q. Zhu X. Yan H.-B. . (2018). Genetic diversity in echinococcus multilocularis from the plateau vole and plateau pika in Jiuzhi County, Qinghai Province, China. Front. Microbiol. 9. doi: 10.3389/fmicb.2018.02632, PMID: 30455674 PMC6230927

[B66] Lundström-Stadelmann B. Rostami A. Frey C. Torgerson P. Riahi S. Bagheri K. . (2025). Human alveolar echinococcosis-global, regional, and national annual incidence and prevalence rates. Clin. Microbiol. infection 31, 1139–1145. doi: 10.1016/j.cmi.2025.01.034, PMID: 40054771

[B67] Ma J. Wang H. Lin G. Craig P. Ito A. Cai Z. . (2012). Molecular identification of Echinococcus species from eastern and southern Qinghai, China, based on the mitochondrial cox1 gene. Parasitol. Res. 111, 179–184. doi: 10.1007/s00436-012-2815-z, PMID: 22258080

[B68] Martini M. Dumendiak S. Gagliardo A. Ragazzini F. La Rosa L. Giunchi D. . (2022). Echinococcus multilocularis and other taeniid metacestodes of muskrats in Luxembourg: prevalence, risk factors, parasite reproduction, and genetic diversity. Pathogens 11, 1414. doi: 10.3390/pathogens11121414, PMID: 36558748 PMC9781964

[B69] Maxam A. Gilbert W. (1977). A new method for sequencing DNA. Proc. Natl. Acad. Sci. U.S.A. 74, 560–564. doi: 10.1073/pnas.74.2.560, PMID: 265521 PMC392330

[B70] McNulty S. Mann P. Robinson J. Duncavage E. Pfeifer J. (2020). Impact of reducing DNA input on next-generation sequencing library complexity and variant detection. J. Mol. diagnostics 22, 720–727. doi: 10.1016/j.jmoldx.2020.02.003, PMID: 32142899

[B71] Mohammadi M. Harandi M. (2024). Revisiting genetic diversity in Echinococcus multilocularis, the role for EmsB microsatellite: A commentary. Infect. Genet. Evol. 119, 105580. doi: 10.1016/j.meegid.2024.105580, PMID: 38431092

[B72] Nakao M. Li T. Han X. Ma X. Xiao N. Qiu J. . (2010a). Genetic polymorphisms of Echinococcus tapeworms in China as determined by mitochondrial and nuclear DNA sequences. Int. J. Parasitol. 40, 379–385. doi: 10.1016/j.ijpara.2009.09.006, PMID: 19800346 PMC2823955

[B73] Nakao M. Sako Y. Ito A. (2003). Isolation of polymorphic microsatellite loci from the tapeworm Echinococcus multilocularis. Infection Genet. Evol. 3, 159–163. doi: 10.1016/S1567-1348(03)00070-4, PMID: 14522179

[B74] Nakao M. Xiao N. Okamoto M. Yanagida T. Sako Y. Ito A. (2009). Geographic pattern of genetic variation in the fox tapeworm Echinococcus multilocularis. Parasitol. Int. 58, 384–389. doi: 10.1016/j.parint.2009.07.010, PMID: 19651237

[B75] Nakao M. Yanagida T. Okamoto M. Knapp J. Nkouawa A. Sako Y. . (2010b). State-of-the-art Echinococcus and Taenia: phylogenetic taxonomy of human-pathogenic tapeworms and its application to molecular diagnosis. Infect. Genet. Evol. 10, 444–452. doi: 10.1016/j.meegid.2010.01.011, PMID: 20132907

[B76] Nakao M. Yokoyama N. Sako Y. Fukunaga M. Ito A. (2002). The complete mitochondrial DNA sequence of the cestode Echinococcus multilocularis (Cyclophyllidea: Taeniidae). Mitochondrion 1, 497–509. doi: 10.1016/S1567-7249(02)00040-5, PMID: 16120302

[B77] Nikendei C. Greinacher A. Cranz A. Friederich H.-C. Stojkovic M. Berkunova A. (2023). Understanding Alveolar echinococcosis patients’ psychosocial burden and coping strategies-A qualitative interview study. PLoS Negl. Trop. Dis. 17, e0011467. doi: 10.1371/journal.pntd.0011467, PMID: 37540639 PMC10403068

[B78] Okamoto M. Bessho Y. Kamiya M. Kurosawa T. Horii T. (1995). Phylogenetic relationships within Taenia taeniaeformis variants and other taeniid cestodes inferred from the nucleotide sequence of the cytochrome c oxidase subunit I gene. Parasitol.Res. 81, 451–458. doi: 10.1007/BF00931785, PMID: 7567901

[B79] Okamoto M. Oku Y. Kurosawa T. Kamiya M. (2007). Genetic uniformity of Echinococcus multilocularis collected from different intermediate host species in Hokkaido, Japan. J. Vet. Med. Sci. 69, 159–163. doi: 10.1292/jvms.69.159, PMID: 17339760

[B80] Oksanen A. Siles-Lucas M. Karamon J. Possenti A. Conraths F. Romig T. . (2016). The geographical distribution and prevalence of Echinococcus multilocularis in animals in the European Union and adjacent countries: a systematic review and meta-analysis. Parasit Vectors 9, 519. doi: 10.1186/s13071-016-1746-4, PMID: 27682156 PMC5039905

[B81] Pedroza Matute S. Iyavoo S. (2025). Implementation of NGS and SNP microarrays in routine forensic practice: opportunities and barriers. BMC Genomics 26, 541. doi: 10.1186/s12864-025-11723-6, PMID: 40437376 PMC12117683

[B82] Petavy A. Tenora F. Deblock S. Sergent V. (2000). Echinococcus multilocularis in domestic cats in France: A potential risk factor for alveolar hydatid disease contamination in humans. Veterinary Parasitol. 87, 151–156. doi: 10.1016/S0304-4017(99)00181-8, PMID: 10622606

[B83] Phan L. Zhang H. Wang Q. Villamarin R. Hefferon T. Ramanathan A. . (2025). The evolution of dbSNP: 25 years of impact in genomic research. Nucleic Acids Res. 53, D925–D931. doi: 10.1093/nar/gkae977, PMID: 39530225 PMC11701571

[B84] Polish L. O’Connell E. Barth T. Gottstein B. Zajac A. Gibson P. . (2022). European haplotype of echinococcus multilocularis in the United States. N Engl. J. Med. 387, 1902–1904. doi: 10.1056/NEJMc2210000, PMID: 36383717 PMC10072850

[B85] Rachel F. Luttermann C. Höper D. Conraths F. Dapprich J. Maksimov P. (2025). Typing of Echinococcus multilocularis by Region-Specific Extraction and Next-Generation Sequencing of the mitogenome. Front. Microbiol. 16. doi: 10.3389/fmicb.2025.1535628, PMID: 40092033 PMC11906691

[B86] Rausch R. (1967). “ On the ecology and distribution of echinococcus spp,” in (Cestoda: taeniidae) and Characteristics of Develpment in the Intermediate Host. Ed. Harold W. (Paris, France: Manter Laboratory of Parasitology: Faculty and Staff Publications). 42 (1), 19–63 10.1051/parasite/1967421019 6076198

[B87] Rinder H. Rausch R. Takahashi K. Kopp H. Thomschke A. Löscher T. (1997). Limited range of genetic variation in Echinococcus multilocularis. J. Parasitol. 83, 1045–1050. doi: 10.2307/3284359, PMID: 9406776

[B88] Romig T. Deplazes P. Jenkins D. Giraudoux P. Massolo A. Craig P. . (2017). “ Chapter five - ecology and life cycle patterns of echinococcus species,” in Echinococcus and Echinococcosis, Part A. Eds. Thompson R. C. A. Deplazes P. Lymbery A. J. (London, United Kingdom: Academic Press), 213–314., PMID: 10.1016/bs.apar.2016.11.002 28131364

[B89] Romig T. Wassermann M. (2024). Echinococcus species in wildlife. Int. J. Parasitol. Parasites Wildl 23, 100913. doi: 10.1016/j.ijppaw.2024.100913, PMID: 38405672 PMC10884515

[B90] Sacheli R. Knapp J. Pholien C. Egrek S. Léonard P. Giot J.-B. . (2023). Genetic diversity of Echinococcus multilocularis specimens isolated from Belgian patients with alveolar echinococcosis using EmsB microsatellites analysis. Infect. Genet. Evol. 116, 105531. doi: 10.1016/j.meegid.2023.105531, PMID: 37992792

[B91] Saiki R. Scharf S. Faloona F. Mullis K. Horn G. Erlich H. . (1985). Enzymatic amplification of beta-globin genomic sequences and restriction site analysis for diagnosis of sickle cell anemia. Science 230, 1350–1354. doi: 10.1126/science.2999980, PMID: 2999980

[B92] Sanger F. Coulson A. Barrell B. Smith A. Roe B. (1980). Cloning in single-stranded bacteriophage as an aid to rapid DNA sequencing. J. Mol. Biol. 143, 161–178. doi: 10.1016/0022-2836(80)90196-5, PMID: 6260957

[B93] Sanger F. Nicklen S. Coulson A. (1977). DNA sequencing with chain-terminating inhibitors. Proc. Natl. Acad. Sci. U.S.A. 74, 5463–5467. doi: 10.1073/pnas.74.12.5463, PMID: 271968 PMC431765

[B94] Santa M. Rezansoff A. Chen R. Gilleard J. Musiani M. Ruckstuhl K. . (2021). Deep amplicon sequencing highlights low intra-host genetic variability of Echinococcus multilocularis and high prevalence of the European-type haplotypes in coyotes and red foxes in Alberta, Canada. PLoS Negl. Trop. Dis. 15, e0009428. doi: 10.1371/journal.pntd.0009428, PMID: 34038403 PMC8153462

[B95] Santa M. Umhang G. Klein C. Grant D. Ruckstuhl K. Musiani M. . (2023). It’s a small world for parasites: evidence supporting the North American invasion of European Echinococcus multilocularis. Proc. Biol. Sci. 290, 20230128. doi: 10.1098/rspb.2023.0128, PMID: 36883278 PMC9993045

[B96] Santoro A. Santolamazza F. Cacciò S. La Rosa G. Antolová D. Auer H. . (2024). Mitochondrial genetic diversity and phylogenetic relationships of Echinococcus multilocularis in Europe. Int. J. Parasitol 54, 233–245. doi: 10.1016/j.ijpara.2024.01.003, PMID: 38246405

[B97] Satam H. Joshi K. Mangrolia U. Waghoo S. Zaidi G. Rawool S. . (2023). Next-generation sequencing technology: current trends and advancements. Biology 12, 997. doi: 10.3390/biology12070997, PMID: 37508427 PMC10376292

[B98] Satam H. Joshi K. Mangrolia U. Waghoo S. Zaidi G. Rawool S. . (2024). Correction: Satam et al. Next-Generation Sequencing Technology: Current Trends and Advancements. Biology 12, 997. doi: 10.3390/biology13050286, PMID: 37508427 PMC10376292

[B99] Schneider C. Kratzer W. Binzberger A. Schlingeloff P. Baumann S. Romig T. . (2023). Echinococcus multilocularis and other zoonotic helminths in red foxes (Vulpes vulpes) from a southern German hotspot for human alveolar echinococcosis. Parasit Vectors 16, 425. doi: 10.1186/s13071-023-06026-2, PMID: 37980538 PMC10657614

[B100] Schurer J. Gesy K. Elkin B. Jenkins E. (2014). Echinococcus multilocularis and Echinococcus canadensis in wolves from western Canada. Parasitology 141, 159–163. doi: 10.1017/S0031182013001716, PMID: 24135428

[B101] Shang J.-Y. Zhang G.-J. Liao S. Yu W.-J. He W. Wang Q. . (2021). Low genetic variation in Echinococcus multilocularis from the Western Sichuan Plateau of China revealed by microsatellite and mitochondrial DNA markers. Acta Tropica 221, 105989. doi: 10.1016/j.actatropica.2021.105989, PMID: 34058159

[B102] Simoncini A. Massolo A. (2024). Multiscale ecological drivers of Echinococcus multilocularis spatial distribution in wild hosts: A systematic review. Food Waterborne Parasitol. 34, e00216. doi: 10.1016/j.fawpar.2023.e00216, PMID: 38152424 PMC10749871

[B103] Šnábel V. Antolová D. Cavallero S. D’Amelio S. (2020). On the geographic genetic variants of the cestode Echinococcus multilocularis with reference to the original descriptions from Bowles et al., (1992) and Bowles and McManus, (1993), and their use. Parasitol. Int. 75, 102039. doi: 10.1016/j.parint.2019.102039, PMID: 31843686

[B104] Spotin A. Boufana B. Ahmadpour E. Casulli A. Mahami-Oskouei M. Rouhani S. . (2018). Assessment of the global pattern of genetic diversity in Echinococcus multilocularis inferred by mitochondrial DNA sequences. Veterinary Parasitol. 262, 30–41. doi: 10.1016/j.vetpar.2018.09.013, PMID: 30389009

[B105] Staubach C. Thulke H. Tackmann K. Hugh-Jones M. Conraths F. (2001). Geographic information system-aided analysis of factors associated with the spatial distribution of Echinococcus multilocularis infections of foxes. Am. J. Trop. Med. Hygiene 65, 943–948. doi: 10.4269/ajtmh.2001.65.943, PMID: 11792003

[B106] Tajima F. (1989). Statistical method for testing the neutral mutation hypothesis by DNA polymorphism. Genetics 123, 585–595. doi: 10.1093/genetics/123.3.585, PMID: 2513255 PMC1203831

[B107] Thompson R. (2017). “ Biology and systematics of echinococcus,” in Echinococcus and Echinococcosis, Part A. Eds. Thompson R. C. A. Deplazes P. Lymbery A. J. (London, United Kingdom: Academic Press), 65–109.

[B108] Thompson R. (2020). The molecular epidemiology of echinococcus infections. Pathogens 9, 1–9. doi: 10.3390/pathogens9060453, PMID: 32521787 PMC7350326

[B109] Thompson R. Deplazes P. Eckert J. (2003). Observations on the development of echinococcus multilocularis in cats. J. Parasitol. 89, 1086–1088. doi: 10.1645/GE-3150RN, PMID: 14640069

[B110] Thompson R. Kapel C. Hobbs R. Deplazes P. (2006). Comparative development of Echinococcus multilocularis in its definitive hosts. Parasitology 132, 709–716. doi: 10.1017/S0031182005009625, PMID: 16420728

[B111] Uakhit R. Smagulova A. Syzdykova A. Abdrakhmanov S. Kiyan V. (2022). Genetic diversity of Echinococcus spp. in wild carnivorous animals in Kazakhstan. Vet. World 15, 1489–1496. doi: 10.14202/vetworld.2022.1489-1496, PMID: 35993082 PMC9375211

[B112] Umhang G. Bastien M. Bastid V. Poulle M.-L. Boué F. (2022). High variability in the number of E. multilocularis eggs in cat feces collected in the field. Parasitol. Int. 89, 102583. doi: 10.1016/j.parint.2022.102583, PMID: 35398276

[B113] Umhang G. Demerson J.-M. Legras L. Boucher J.-M. Peytavin de Garam C. Bastid V. . (2021a). Rodent control programmes can integrate Echinococcus multilocularis surveillance by facilitating parasite genotyping: the case of Arvicola terrestris voles screening in France. Z. für Parasitenkunde 120, 1903–1908. doi: 10.1007/s00436-021-07126-7, PMID: 33742248

[B114] Umhang G. Forin-Wiart M.-A. Hormaz V. Caillot C. Boucher J.-M. Poulle M.-L. . (2015). Echinococcus multilocularis detection in the intestines and feces of free-ranging domestic cats (Felis s. catus) and European wildcats (Felis s. silvestris) from northeastern France. Veterinary Parasitol. 214, 75–79. doi: 10.1016/j.vetpar.2015.06.006, PMID: 26206606

[B115] Umhang G. Karamon J. Hormaz V. Knapp J. Cencek T. Boué F. (2017). A step forward in the understanding of the presence and expansion of Echinococcus multilocularis in Eastern Europe using microsatellite EmsB genotyping in Poland. Infect. Genet. Evol. 54, 176–182. doi: 10.1016/j.meegid.2017.07.004, PMID: 28688974

[B116] Umhang G. Knapp J. Hormaz V. Raoul F. Boué F. (2014). Using the genetics of Echinococcus multilocularis to trace the history of expansion from an endemic area. Infection Genet. Evol. 22, 142–149. doi: 10.1016/j.meegid.2014.01.018, PMID: 24468327

[B117] Umhang G. Knapp J. Wassermann M. Bastid V. Peytavin de Garam C. Boué F. . (2021b). Asian admixture in european echinococcus multilocularis populations: new data from Poland comparing emsB microsatellite analyses and mitochondrial sequencing. Front. Veterinary Sci. 7. doi: 10.3389/fvets.2020.620722, PMID: 33521093 PMC7843918

[B118] Valot B. Knapp J. Umhang G. Grenouillet F. Millon L. (2015). Genomic characterization of EmsB microsatellite loci in Echinococcus multilocularis. Infection Genet. Evol. 32, 338–341. doi: 10.1016/j.meegid.2015.03.040, PMID: 25847697

[B119] van Herwerden L. Gasser R. Blair D. (2000). ITS-1 ribosomal DNA sequence variants are maintained in different species and strains of Echinococcus. Int. J. Parasitol. 30, 157–169. doi: 10.1016/S0020-7519(00)00002-3, PMID: 10704599

[B120] Virchow R. (1855). Die multiloculäre, ulcerirende Echinokokkengeschwulst der Leber. Verhandlungen der Physikalisch-Medizinischen Gesellschaft zu Würzburg 6, 84–95. Available online at: http://publikationen.ub.uni-frankfurt.de/frontdoor/index/index/docId/13560.

[B121] Vogel H. (1957). Über den Echinococcus multilocularis Süddeutschlands I. Das Bandwurmstadium von Stämmen menschlicher und tierischer Herkunft (Echinococcus multilocularis in South Germany. I. The tapeworm stage of strains from humans and animals). Z. für Tropenmedizin und Parasitologie 8, 404–454. 13496995

[B122] Vogel M. Müller N. Gottstein B. Flury K. Eckert J. Seebeck T. (1990). Echinococcus multilocularis: characterization of a DNA probe. Acta Tropica 48, 109–116. doi: 10.1016/0001-706x(90)90050-a, PMID: 1980566

[B123] von Nickisch-Rosenegk M. Lucius R. Loos-Frank B. (1999). Contributions to the phylogeny of the cyclophyllidea (Cestoda) inferred from mitochondrial 12S rDNA. J. Mol. Evol. 48, 586–596. doi: 10.1007/PL00006501, PMID: 10198124

[B124] Vuitton D. Demonmerot F. Knapp J. Richou C. Grenouillet F. Chauchet A. . (2015). Clinical epidemiology of human AE in Europe. Veterinary Parasitol. 213, 110–120. doi: 10.1016/j.vetpar.2015.07.036, PMID: 26346900

[B125] Vuitton D. McManus D. Rogan M. Romig T. Gottstein B. Naidich A. . (2020). International consensus on terminology to be used in the field of echinococcoses. Parasite 27, 1–41. doi: 10.1051/parasite/2020024, PMID: 32500855 PMC7273836

[B126] Wang B. Zhao L. Ban W. Zhang X. Quan C. Teliewuhan M. . (2024). Investigation and genetic polymorphism analysis of rodents infected with Echinococcus in Ili Prefecture, Xinjiang Uygur Autonomous Region, China. Front. Cell. Infect. Microbiol. 14. doi: 10.3389/fcimb.2024.1433359, PMID: 39185087 PMC11341461

[B127] Wen H. Vuitton L. Tuxun T. Li J. Vuitton D. Zhang W. . (2019). Echinococcosis: advances in the 21st century. Clin. Microbiol. Rev. 32, 1–39. doi: 10.1128/CMR.00075-18, PMID: 30760475 PMC6431127

[B128] Woolsey I. Miller A. (2021). Echinococcus granulosus sensu lato and Echinococcus multilocularis: A review. Res. Veterinary Sci. 135, 517–522. doi: 10.1016/j.rvsc.2020.11.010, PMID: 33246571

